# A Premature Rabbit Kitten Model for Evaluation of Bronchopulmonary Dysplasia Therapies Reproduces Myeloperoxidase-Associated Pathology and Therapeutic Responses

**DOI:** 10.3390/ijms27167284

**Published:** 2026-08-15

**Authors:** Carlos Dounce, Xigang Jing, Keguo Li, Deron Jones, Tzong-Jin Wu, Adeleye J. Afolayan, Girija Ganesh Konduri, Billy W. Day, Kirkwood A. Pritchard, Stephen Naylor, Ru-Jeng Teng

**Affiliations:** 1 Division of Neonatology, Department of Pediatrics, Medical College of Wisconsin, Wauwatosa, WI 53226, USA; cdounce@mcw.edu (C.D.); xjing@mcw.edu (X.J.); twu@mcw.edu (T.-J.W.); aafolaya@mcw.edu (A.J.A.); gkonduri@mcw.edu (G.G.K.); 2 Division of Pediatric Surgery, Department of Surgery, Medical College of Wisconsin, Wauwatosa, WI 53122, USA; kli@mcw.edu (K.L.); djones@mcw.edu (D.J.);; 3 ReNeuroGen LLC, Milwaukee, WI 53122, USA; billy.day@rngen.com (B.W.D.); snaylor@rngen.com (S.N.)

**Keywords:** bronchopulmonary dysplasia, premature rabbit kitten, myeloperoxidase, N-acetyl-lysyltyrosylcysteine amide, hyperoxia, developmental lung injury, therapeutic evaluation

## Abstract

Myeloperoxidase (MPO) plays a significant role in the development and progression of bronchopulmonary dysplasia (BPD). The neonatal rat model has shown that the tripeptide N-acetyl-lysyltyrosylcysteine amide (KYC) attenuates hyperoxia-induced BPD-like injury, in part by reducing MPO-mediated toxic oxidants and additional cytoprotective mechanisms. However, the rat model does not fully replicate the key characteristics of surfactant deficiency and immature antioxidant capacity in premature human neonates. In contrast, premature rabbit kittens share key features with those of premature human neonates. For this study, premature rabbit kittens delivered at 29 days of gestation (term is 31 days) were exposed to 95% oxygen for 7 days. Hyperoxia caused a BPD-like phenotype characterized by increased MPO-positive inflammatory cell infiltration, elevated MPO expression, alveolar simplification, septal wall thickening, and pulmonary arterial medial wall thickening. Daily intraperitoneal KYC administration attenuated lung inflammatory cell infiltration, reduced MPO expression, improved alveolar morphometric indices, and mitigated pulmonary arterial medial wall thickening compared with phosphate-buffered saline treatment. There were no significant differences in weight changes or survival between the groups. These findings demonstrate that premature rabbit kittens provide a useful developmental model for the evaluation of candidate BPD therapies and reproduce MPO-associated pathological and therapeutic responses previously observed in neonatal rat studies.

## 1. Introduction

Bronchopulmonary dysplasia (BPD) is the most common lung complication of prematurity, with more than 10,000 newly diagnosed cases annually in the USA [1]. Other than prolonged oxygen dependency, BPD is also associated with a poor neurodevelopmental outcome, frequent post-discharge rehospitalization [2], high blood pressure [3], reactive airway disease [4], poor exercise endurance [5], and early onset of chronic obstructive pulmonary disease [6]. Roughly one-fourth of the moderate-to-severe BPD infants also develop pulmonary hypertension, which increases the odds of death by several folds [7]. Most importantly, the financial burden on the family and the socioeconomic impact of BPD are extremely high [8].

Although vitamin A treatment [9] and caffeine therapy [10] have been shown to reduce BPD incidence, the prevalence of BPD has not decreased for decades [11]. Improved neonatal care, with an increased survival rate among periviable neonates [12], paradoxically contributes to the unwavering BPD prevalence. Treatment that can either prevent the development of BPD or mitigate the severity of BPD can benefit these at-risk premature infants. Prematurity with surfactant deficiency and underdeveloped antioxidative capacity is the root cause of BPD. Oxidative stress, infection, and trauma from mechanical ventilation further suppress the growth potential of the premature lungs. In fact, clinical studies have shown that oxidative stress and inflammation not only activate each other but are also closely associated with preterm birth [13]. Life-saving mechanical ventilation further aggravates oxidative stress [14] and innate lung inflammation [15].

BPD is a multifactorial disorder with its mechanisms remaining incompletely understood. Rodent pups exposed to a high-oxygen environment (hyperoxia, HOX) are the most commonly used animal model for studying the mechanisms underlying BPD development [16]. Alveolar simplification and pruning of the lung microvasculature under HOX are morphological changes that are reminiscent of human BPD. We have repeatedly demonstrated that myeloperoxidase (MPO) plays a crucial role in the initiation and progression of the BPD phenotype in rat pups [17]. The increased MPO level in the lung subsequently elicits a series of signaling pathways, including the unfolded protein response (UPR), autophagy, damage-associated molecular patterns (DAMP) signaling, cell death, mitochondrial dysfunction, and cellular senescence, thereby forming a self-perpetuating destructive cycle that recruits more inflammatory cells to damage the neonatal lungs [18].

The importance of MPO in BPD is further supported by our studies showing that the peptide-based agent N-acetyl-lysyltyrosylcysteine amide (KYC) attenuates BPD severity by reducing MPO-associated oxidative injury, innate inflammation, MPO expression, UPR [19], cell death, and cellular senescence in HOX rat pup lungs [20]. KYC offers additional layers of protection, including stabilizing Nrf2 to activate antioxidative enzymes, inhibiting Hmgb1-mediated DAMP signaling [21] and preserving type 2 alveolar cells [20]. We have provided evidence that KYC protects the neonatal lung through multiple mechanisms; thus, we consider KYC to behave as a Systems Pharmacotherapy Agent (SPTA) [18]. Unlike classical MPO inhibitors, KYC reduces MPO-mediated formation of toxic oxidants while preserving additional MPO-dependent protective activities. These unique properties distinguish KYC from classical MPO inhibitors. Our series of studies has raised the possibility that MPO is both an injury mediator and a therapeutic target for BPD.

Although we [18] and others [16] have used the rat as the animal model to understand the pathophysiology of BPD, we all encountered some barriers including sufficient surfactants at birth [22], mature antioxidative capacity and rapid upregulation of antioxidant enzymes [23], and immature MPO response immediately after birth [24], which prevent the findings from being directly translated to human premature neonates. In contrast, premature rabbit kittens are well-documented to be born with surfactant insufficiency [25] and exhibit an immature antioxidant response at birth [26,27], supporting their use as a developmentally relevant model for selected features of premature human lung injury [28]. They develop pulmonary hypertension after exposure to a high-oxygen environment for seven days, which resembles what occurs in moderate-to-severe BPD infants [29]. This model has been used successfully to demonstrate the therapeutic potential of several medications in attenuating the severity of BPD [30,31,32]. However, the MPO-targeted therapeutic strategy has not been explored in a premature rabbit model.

Rabbits can provide advantages for studying MPO biology because their neutrophils exhibit higher MPO activity and a stronger response to stimulation than those of rats [33]. The developmental similarities between premature rabbit kittens and premature human neonates may provide broader value in evaluating candidate BPD therapies. In addition to developmental similarities, rabbit neutrophils exhibit MPO activity and responsiveness that may more closely approximate human biology than that observed in rodents [24]. This characteristic may increase the value of the premature rabbit kitten model for studying MPO-associated injury pathways and evaluating candidate therapies. The key novelty of this study lies not in establishing a new model of BPD, but in using a developmentally relevant premature rabbit kitten model. This model is employed to determine whether the MPO-associated pathology and KYC-associated therapeutic responses previously observed in neonatal rat pups can be replicated. It is characterized by surfactant insufficiency, immature antioxidant capacity, and a more robust neutrophil/MPO response. To our knowledge, this is the first time an MPO-targeted therapeutic approach has been assessed in this premature rabbit BPD model.

## 2. Results

In general, the current study has demonstrated findings that are parallel to prior observations from neonatal rat pup studies. Seven kittens from one doe were randomly assigned to NOX (N = 3) and HOX (N = 4) to confirm that similar phenotypical changes in rat pups can be reproduced in the rabbit model, not the main therapeutic study. One kitten in HOX died. Fifty-five kittens from six does survived the first 60 min and were randomized into four groups (NOX-PBS 14; NOX-KYC 12; HOX-PBS 16; HOX-KYC 13) for the subsequent experiments; 10 died before P7.

### 2.1. Hyperoxia Causes Inflammatory Cell Infiltration and Alveolar Simplification in the Lungs of Premature Rabbits

**Figure 1:**
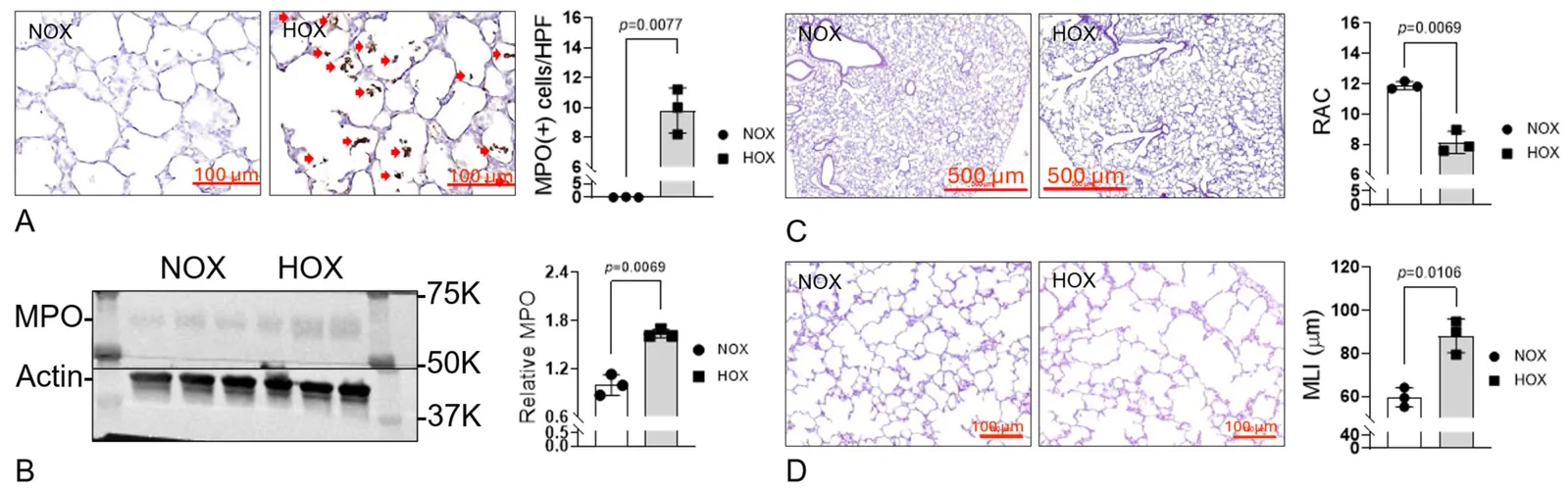
Exposure to hyperoxia (HOX) induces inflammation and leads to the simplification of alveoli in the lungs of premature rabbit kittens. (**A**) There is a significant increase in the number of myeloperoxidase (MPO)-positive myeloid cells as compared to normoxia (NOX) exposure. (**B**) MPO levels in lung lysates are notably elevated. (**C**) The radial alveolar count (RAC) has diminished. (**D**) The mean linear intercept (MLI) has increased. *p*-values obtained by the unpaired Welch’s test, N = 3. Red arrow stands for inflammatory cell.

Consistent with our findings from studies of BPD in rats, we observe a significant increase in MPO (+) myeloid cell infiltration in the lungs of premature rabbits exposed to HOX. Specifically, the HOX count was 9.8 ± 1.5 per high-power field (HPF) compared to only 0.1 ± 0.1 per HPF under normoxic (NOX) conditions, as illustrated in Figure 1A. Furthermore, MPO expression was notably higher, registering at 1.6 ± 0.1 versus 1.0 ± 0.1, as shown in Figure 1B. Additionally, we noted a significant reduction in the radial alveolar count (RAC), from 11.9 ± 0.3 to 8.1 ± 0.7, as shown in Figure 1C. Alongside this, the mean linear intercept (MLI) increased to 88.1 ± 7.9 µm compared to 59.6 ± 4.5 µm, as shown in Figure 1D. These findings collectively suggest that HOX exposure leads to alveolar simplification.

### 2.2. N-Acetyl-Lysyltyrosylcysteine Amide (KYC) Attenuates HOX-Induced Lung Inflammation

**Figure 2:**
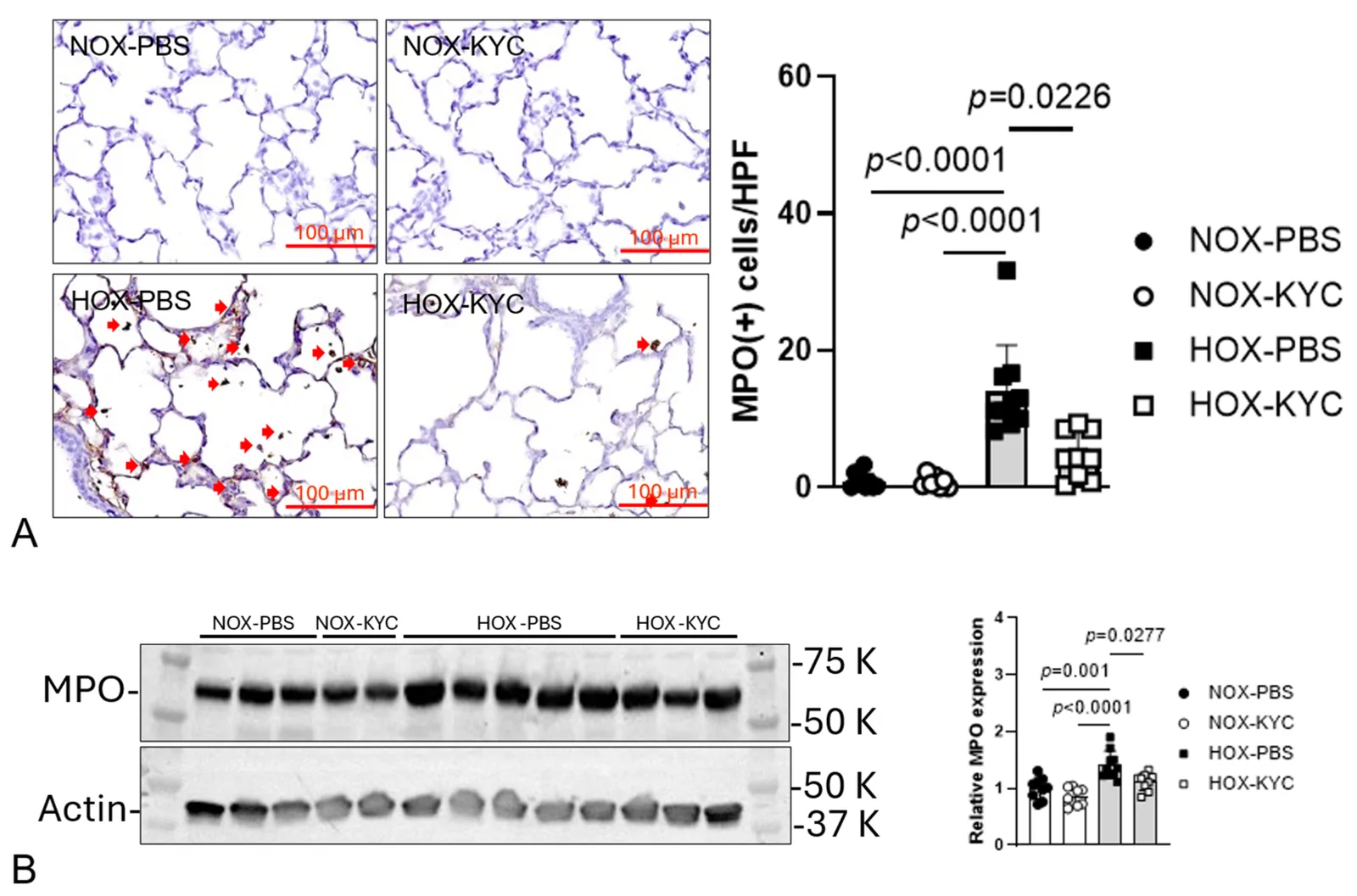
Daily KYC treatment effectively mitigates lung inflammation in premature rabbit kittens exposed to HOX. (**A**) KYC significantly reduces inflammatory cell infiltration in the lungs of HOX-exposed kittens. (**B**) KYC substantially reduces MPO expression in the lungs exposed to HOX. NOX-PBS: kittens raised in NOX and treated with phosphate-buffered saline (PBS); *n* = 9; NOX-KYC: kittens raised in NOX and treated with KYC; *n* = 8; HOX-PBS: kittens raised in HOX and treated with PBS; *n* = 10; HOX-KYC: kittens raised in HOX and treated with KYC; *n* = 8. *p*-values were determined by the Kruskal–Wallis test with post hoc Conover–Iman test, based on the skewed data distribution. Red arrow stand for inflammatory cell.

As we have reported in experiments with rat pups, daily KYC treatment in HOX-exposed premature rabbit kittens significantly reduced the infiltration of MPO (+) myeloid cells. However, the level of myeloid cell infiltration remained considerably higher than that observed in the two NOX groups, with counts of 0.9 ± 1.2/HPF, 0.8 ± 0.9/HPF, 14.0 ± 6.8/HPF, and 4.7 ± 3.4/HPF for NOX-PBS, NOX-KYC, HOX-PBS, and HOX-KYC, respectively (Figure 2A). The relative levels of MPO exhibited a similar trend, with values of 1.0 ± 0.2, 0.9 ± 0.2, 1.4 ± 0.2, and 1.1 ± 0.2 for NOX-PBS, NOX-KYC, HOX-PBS, and HOX-KYC, respectively (Figure 2B). These findings suggest that KYC effectively attenuates HOX-induced inflammation.

### 2.3. KYC Mitigates HOX-Induced Alveolar Structural Changes

**Figure 3:**
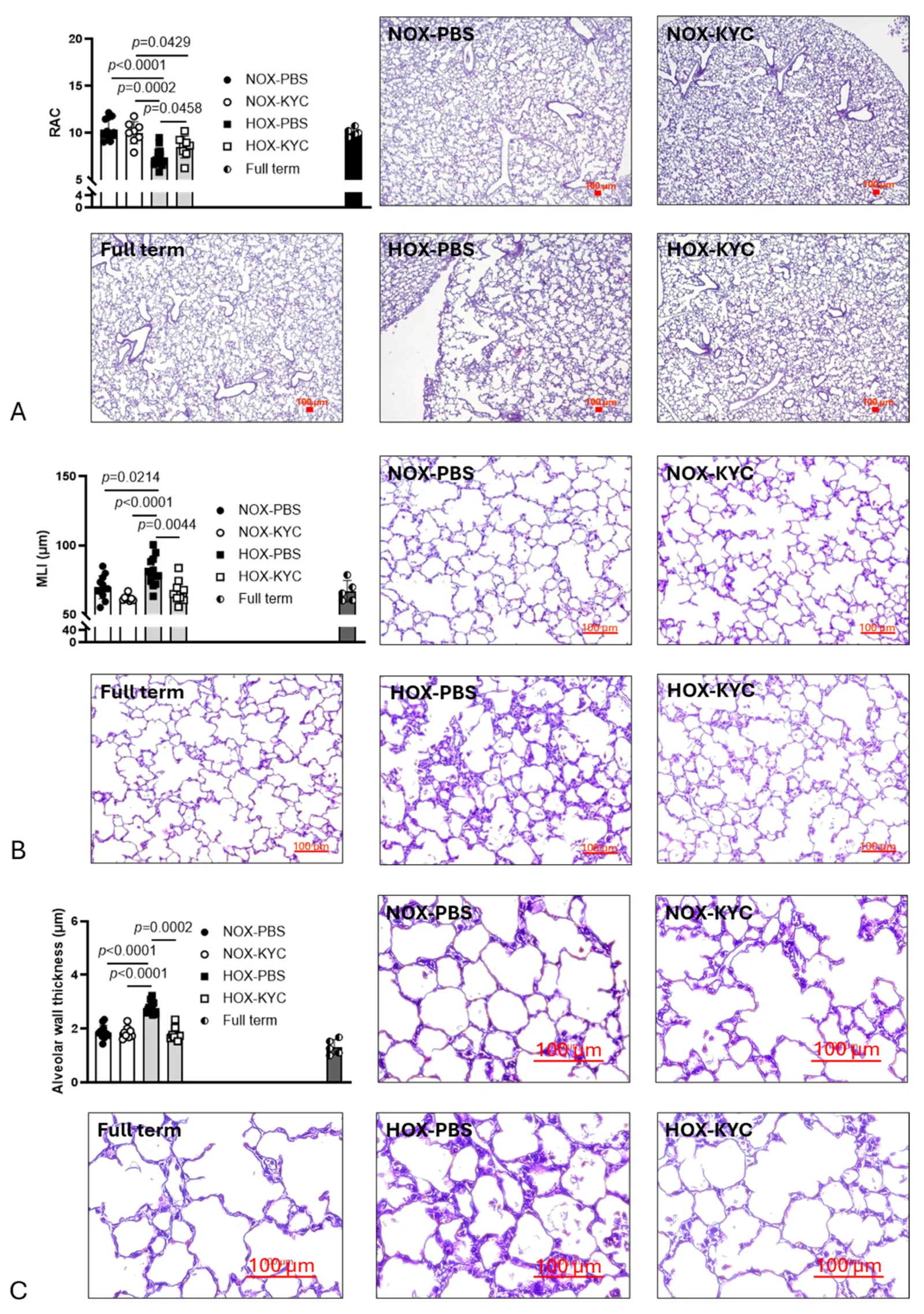
Daily KYC treatment mitigates the structural changes in the alveoli of HOX-exposed premature rabbit kittens. (**A**) HOX exposure decreases radial alveolar count (RAC) in premature lungs. However, daily KYC treatment partially but significantly maintains RAC levels. (**B**) HOX exposure increases the mean linear intercept (MLI), while KYC treatment significantly reverses this effect. (**C**) KYC also significantly reduces HOX-induced septal wall thickening (SWT). Morphometric data from term rabbit kittens (N = 5) are used as a reference. There are no significant differences in RAC or MLI between the full-term and NOX groups; however, SWT in full-term kittens is significantly lower than in both NOX groups.

The morphometric data show that daily KYC treatment in premature rabbit kittens partially reversed alveolar simplification under HOX, with RAC of 10.4 ± 1.1, 10.0 ± 1.2, 7.4 ± 1.0, and 8.5 ± 1.2 for NOX-PBS, NOX-KYC, HOX-PBS, and HOX-KYC, respectively (Figure 3A). KYC partially maintained the volume-to-surface ratio of acinar airspaces with MLI of 69.8 ± 8.4 µm, 61.8 ± 2.2 µm, 80.9 ± 10.3 µm, and 66.7 ± 8.4 µm for NOX-PBS, NOX-KYC, HOX-PBS, and HOX-KYC, respectively (Figure 3B). KYC significantly reduced HOX-induced septal wall thickening (SWT), a structural change believed to improve gas exchange efficiency, with average SWT values of 1.9 ± 0.3 µm, 1.9 ± 0.2 µm, 2.8 ± 0.2 µm, and 1.8 ± 0.2 µm for NOX-PBS, NOX-KYC, HOX-PBS, and HOX-KYC, respectively (Figure 3C). Although there is no difference among full-term, NOX-PBS, and NOX-KYC for RAC and MLI, the average SWT is significantly lower than in NOX-PBS and NOX-KYC, at 1.3 ± 0.3 µm for full-term kittens.

### 2.4. KYC Does Not Affect Survival Rate and Weight Gain

**Figure 4:**
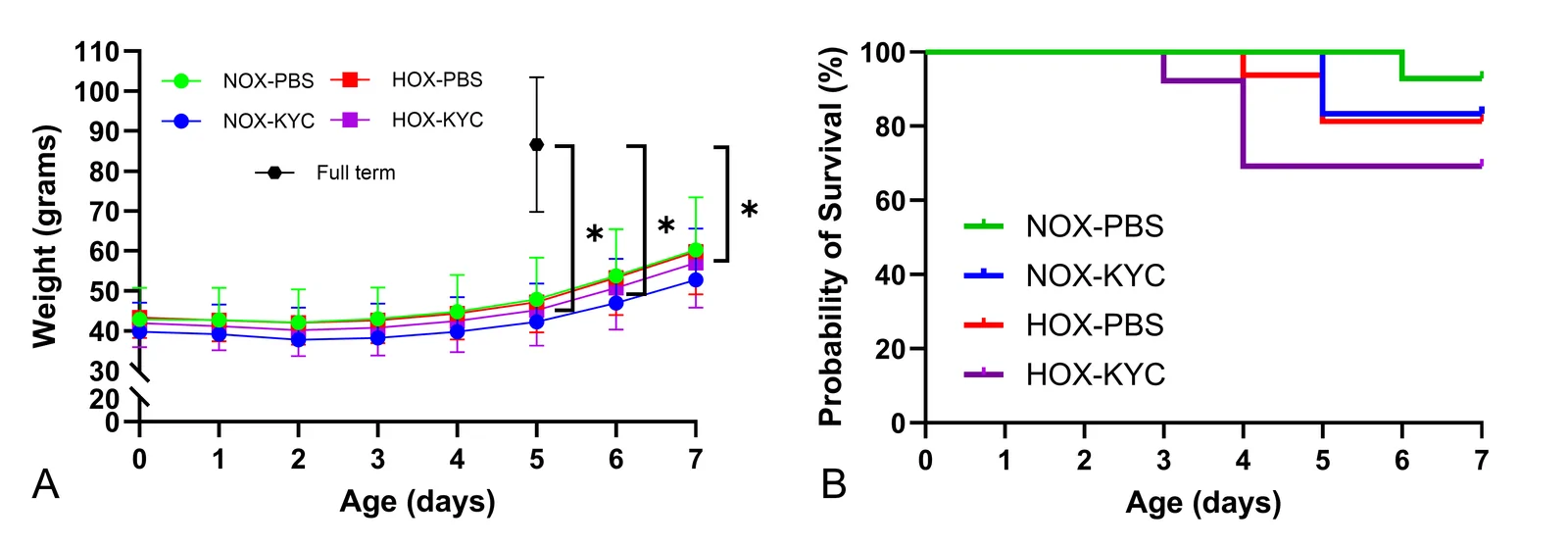
Neither hyperoxia (HOX) exposure nor KYC treatment affects the weight changes or survival rate in premature rabbit kittens. (**A**) There is a wide variation in birth weights due to differences in litter size. There was no significant difference in postnatal weight changes between study groups. The body weights of all four premature groups are significantly lower at postnatal day 7 (P7) than those of full-term kittens of equivalent postconceptional age at P5. (**B**) Although the survival rate of the HOX-KYC seems to be lower than that of other groups, no significant difference in survival rate among the four treatment groups was observed. * *p* < 0.05 according to the Kruskal–Wallis test with post hoc Conover–Iman test.

There was a wide range of birth weights, probably due to variation in litter sizes (N = 2–12). However, with randomization, no significant difference in body weight was detected among the four groups. Kittens regained their birth weight at P3, but their weights at P7 were significantly lower than those of full-term kittens of equivalent postconceptional age at P5 (*p* < 0.05; Figure 4A). The survival rates for the four premature groups ranged from 70% to 93%. Although the survival rate appears lower in the HOX-KYC group, we did not detect a statistically significant difference (*p* = 0.37) among the four treatment groups (Figure 4B).

### 2.5. KYC Attenuates HOX-Induced Medial Wall Thickening of Pulmonary Resistance Arteries

**Figure 5:**
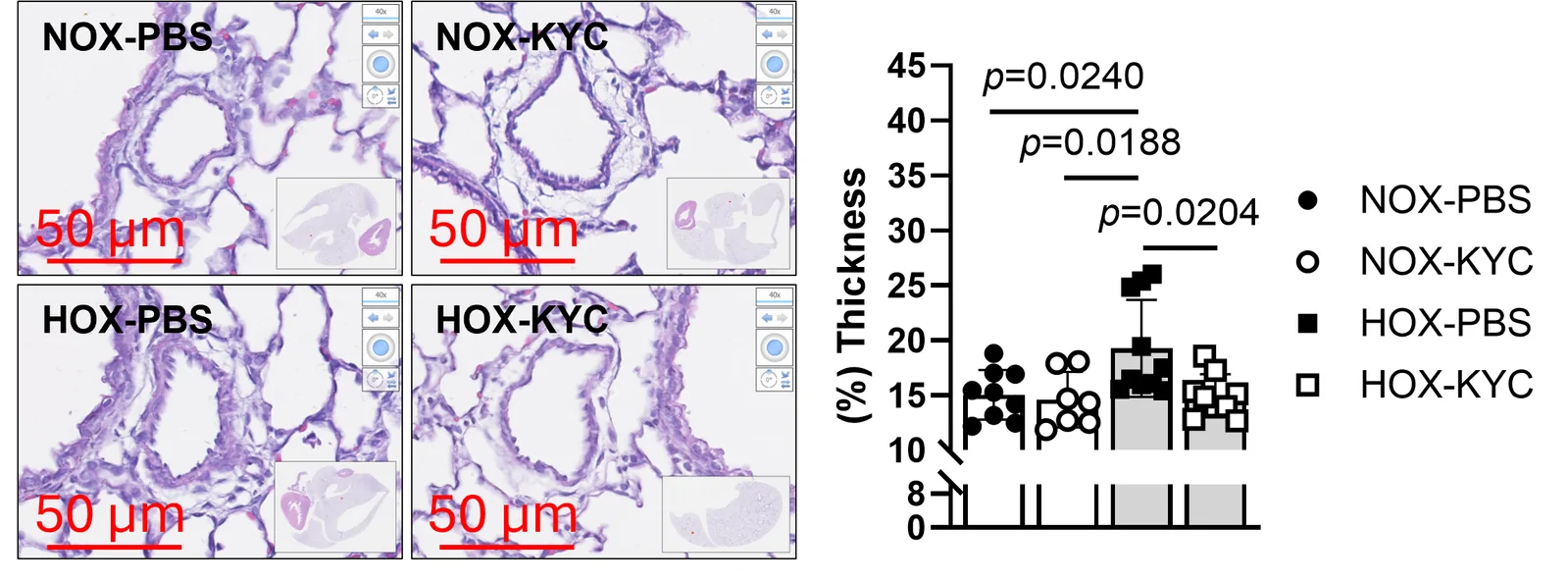
KYC attenuates HOX-induced medial wall thickening of pulmonary resistance arteries in premature rabbit kittens. There is no difference between NOX-PBS, NOX-KYC, and HOX-KYC groups. *p*-values are determined by the Kruskal–Wallis test with post hoc Conover–Iman test.

Medial wall thickness of the pulmonary resistance arteries correlates with the severity and progression of pulmonary hypertension. We detected significant medial wall thickening of the pulmonary resistance arteries in the HOX-PBS group, which KYC treatment mitigated. The thicknesses were 15.1 ± 2.3%, 14.6 ± 2.5%, 19.3 ± 4.4%, and 15.0 ± 2.0% for NOX-PBS, NOX-KYC, HOX-PBS, and HOX-KYC, respectively (Figure 5).

## 3. Discussion

Numerous mechanisms have been identified as playing a role in the development and progression of BPD [13]. Some of these mechanisms create a complex network of interactions that lead to an MPO-mediated, self-perpetuating, destructive cycle in the HOX rat pup model, as we have reported [18]. This finding prompted us to explore the therapeutic potential of MPO inhibitors in safeguarding the growth of premature lungs. Our investigations have centered on the effects of KYC, a peptide-based agent that modulates MPO-associated oxidative injury, within our rat BPD model. These studies have demonstrated that KYC protects neonatal lungs from HOX-induced alveolar simplification. The underlying mechanisms include attenuation of the unfolded protein response (UPR), apoptosis, cellular senescence, mitochondrial dysfunction, DAMP signaling, and enhanced antioxidative response [19,20,21], establishing KYC as a unique SPTA and a promising therapeutic candidate.

Although our previous studies have clearly demonstrated that KYC effectively reduces BPD severity [19,20,21], the rat model lacks several key components of human BPD, such as prematurity, surfactant insufficiency, and reduced antioxidative capacity. The weak and delayed MPO response makes rat pups less suitable for MPO studies [24]. We are concerned about the relevance of our findings in rats to those in human premature neonates. The purpose of this study was to ensure the replicability and reproducibility of findings observed in the rat BPD model using premature rabbit kittens. We are encouraged that the observed morphometric changes support the protective effects of KYC in premature rabbit lungs exposed to HOX.

Our original hypothesis was that KYC attenuates BPD severity by reducing MPO-mediated oxidative injury and suppressing lung inflammation [21]. Given surfactant insufficiency [25] and underdeveloped antioxidative capacity [26,27], we expected inflammation to be more aggressive in the lungs of premature rabbits [34]. This is especially important because the MPO response has been reported to be stronger in rabbits than in rats [24]. If KYC can also suppress the inflammatory response in premature rabbit lungs, this would strengthen the rationale for further translational development. We also used KYC as the therapeutic probe to validate the utility of the premature rabbit model. As we have reported in rat pups, we saw inflammatory cell infiltration and increased MPO expression in HOX rabbit lungs, and KYC treatment successfully reduced lung inflammation. These new data strongly support our hypothesis.

The alveolar simplification under HOX and the increased alveolar complexity by KYC treatment in HOX-exposed premature rabbit lungs successfully reproduce the results from our rat model studies [20,21]. The concordance across species strengthens confidence in the biological significance of the observed effects. These facts reinforce the conclusion that the premature rabbit model resembles several key developmental features of premature human neonates. Surprisingly, we did not observe significant differences in morphometrics, except for the SWT, between NOX-exposed lungs from premature and full-term rabbits with the same postconceptional age. One possible explanation for this lack of difference is that the E29 rabbit lungs are developmentally equivalent to those of moderately premature human neonates and are therefore more resistant to HOX [35]. The higher SWT in NOX-premature kittens than in full-term kittens may reflect developmental processes [30]. In comparison, the high SWT in HOX-exposed kittens may result from inflammation, dysregulated extracellular matrix deposition, or arrested microvascular maturation, a process that KYC mitigates. Because increased SWT is expected to decrease lung compliance and impair diffusion capacity [36], the reduction in SWT observed with KYC treatment would be expected to improve gas exchange efficiency and lung mechanics during the early postnatal period. It will be essential to confirm these changes in lung function in our future explorations.

Our premature rabbit kittens exhibit wide variation in birth weight, which we believe to result from differences in litter size. However, after randomization, we did not observe differences in birth weight or weight change across the four treatment groups. Because postnatal growth restriction is a well-described risk factor for BPD [37], the changes in lung morphometry of our rabbit kittens cannot be explained by the difference in postnatal weight gain. The body weight data show that premature rabbit kittens fail to catch up on intrauterine growth during the study period, a pattern commonly observed in human preterm neonates [38]. Interestingly, this does not seem to affect lung morphometry in premature rabbit kittens when they are nursed in room air. Still, we must keep in mind that E29 rabbit kittens are developmentally equivalent to 28–32-week moderate-premature human neonates [35]. In comparison, the E28 rabbit kittens are better suited for studying extremely premature infants, e.g., 24–28 weeks, who are at much higher risk of BPD. The survival rate for E28 premature rabbit kittens, especially under 95% oxygen, will be the challenge. Regardless, we plan to study the effect of KYC on E28 premature rabbit kittens in our next project.

Pulmonary hypertension is a common morbid complication of BPD with a prevalence of roughly 25%, which has gained much recent attention due to its association with a high mortality rate [39]. A few mechanisms have been reported to contribute to its development, and MPO is one of them [40]. It is believed that MPO activates ROCK signaling to promote the proliferation of vascular smooth muscle cells in pulmonary resistance arteries, resulting in medial wall thickening. Although pulmonary hypertension is a late phenotype in the premature rabbit model [35], we still observe a thicker medial wall of the pulmonary resistance arteries at P7 in HOX-exposed kittens. It is encouraging to see that KYC treatment is associated with reduced medial wall thickening in HOX-exposed premature rabbit kittens. These findings suggest that KYC may attenuate early pulmonary vascular remodeling, although we have not yet measured parameters of pulmonary hypertension. Future investigations, including echocardiography, direct right ventricular pressure measurements, and the Fulton index, will provide a better answer to this observation.

The premature rabbit kitten offers complementary advantages for BPD investigations because it is at a lung developmental stage comparable to that of human premature neonates. Compared to the rat model, premature rabbit kittens have surfactant deficiency, altered vascular tone, and systemic immaturity. Because we can determine the time of birth, we can use them for studying complex BPD phenotypes, including prenatal insults (lipopolysaccharide-induced chorioamnionitis or fetal growth restriction by maternal hypoxia) [36] and postnatal mechanical ventilation [36]. The size of premature rabbits makes intratracheal drug administration, instrumentation, direct lung function testing, and repeat blood sampling possible [37]. Another benefit of using the rabbit model is that the MPO content per neutrophil and the myeloid response are reminiscent of humans [38]. Although the antioxidant capacity is immature in premature rabbits, they can robustly upregulate antioxidant proteins in a few days [26], similarly to that of rodent pups born at term. However, the low survival rate presents significant challenges and necessitates extensive expertise. The primary obstacle to using rabbits in studies is the lack of adequate tools, including antibodies, reagents, comprehensive reference databases, bioinformatics resources, and complete genome annotations. Despite these limitations, premature rabbit kittens have a broader utility for evaluating candidate BPD therapies.

The significance of the current findings lies in their extension of previous observations from term neonatal rat studies to a translational and developmentally relevant model of premature rabbits. The rabbit kittens’ capacity to mimic MPO-associated pathology and therapeutic responses enhances confidence in both the biological importance of MPO and the model’s effectiveness for assessing potential BPD therapies.

## 4. Materials and Methods

KYC was synthesized using Fmoc [*N*-(9-fluorenyl) methoxy-carbonyl] chemistry and prepared and purified as an acetate salt by Biomatik USA, LLC (Wilmington, DE, USA), as previously described [41]. Trifluoroacetic acid (TFA) was removed by dissolving KYC in 6 mM HCl, followed by lyophilization (2–3×). TFA in KYC was <0.1% before use in experiments, as confirmed by NMR [42] at the Biomolecular NMR Facility of MCW. RP-HPLC was used to determine the purity of the KYC. Only batches with purity >98% were used in our studies. KYC was freshly dissolved in phosphate-buffered saline (PBS) at a concentration of 10 mg/mL and filtered through a 0.22 μm syringe filter for injection. Goat anti-MPO antibody (AF3667) from R&D Systems (Minneapolis, MN, USA) was used for immunoblots. Rabbit anti-MPO antibody (EPR17996) for IHC was from Abcam (Waltham, MA, USA). Rabbit anti-β-actin-HRP antibody (D6A8, #12620) was from Cell Signaling Technology (Danvers, MA, USA).

### 4.1. Animal Care

The Medical College of Wisconsin Institutional Animal Care and Use Committee approved the animal protocol (AUA00007966), which conforms to the National Institutes of Health Guide for the Care and Use of Laboratory Animals. Timed-pregnant New Zealand White rabbits (HsdOkd: NZW) were obtained from Inotiv (Madison, WI, USA) and acclimated in our animal care facility for 7 days in a 12 h dark-light cycle, at 21–24 °C and 40–50% humidity, and with free access to chow and water. Cesarean section was performed at E29 (equivalent to human premature neonates at 28–32 weeks) [43] in the early morning under 3% isoflurane anesthesia through a mask with oxygen flow at 2 LPM, and successful anesthesia was confirmed by toe-pinch. A pregnant doe was allowed to carry to term (31 days) and nurse the kittens in room air. The full-term kittens were weighed only once at P5 (the same postconceptional age as prematurely born kittens) before euthanasia to serve as a reference for comparison. The does were euthanized after the cesarean section by exsanguination under isoflurane anesthesia. Due to the variation in litter sizes (N = 2~12/doe), other than the first experiment with one doe to confirm the creation of the phenotype, we ordered two does for each subsequent experiment. The seven kittens were randomly assigned to NOX (N = 3) or HOX (N = 4) for the first experiment. After the initial 30 min stabilization at 35 °C in a room-air incubator, kittens from two pregnant does were randomly assigned to the NOX and HOX groups within each litter and sex stratum. Kittens were assigned to treatment groups using a pre-specified randomization list generated before treatment allocation. Group assignments were made by one investigator not involved in subsequent image acquisition or morphometric analysis. Histological sections were coded before imaging and analysis, and outcome assessment was performed by investigators blinded to treatment group.

Premature rabbit kittens were dried immediately with a prewarmed dry towel to stimulate spontaneous breathing, then transferred to a prewarmed incubator at 34–35 °C with 50% humidity in a TLC-50 Advance (Brinsea^®^ Products, Weston Super Mare, UK). We found that most (>50%) E29 premature rabbit kittens died before P3 if the incubator temperature was less than 30 °C within the first 24 h of life. Oxygen was supplied to the oxygen chamber, which was placed inside the incubator. Oxygen concentrations were continuously monitored with an oxygen sensor (Reming Bioinstruments Co., Redfield, NY, USA). The incubator temperature was decreased by 1 °C each day.

D10W 25 µL/g was given enterally within 30 min via an Argon 1.9F polyurethane catheter to prevent hypoglycemia. Kittens received their first feed with Wombaroo Rabbit Milk Replacer (Wombaroo Passwell, Mount Barker, SA, Australia) at 40 mL/kg after stabilizing in the incubator for 1 h, and the 2nd at 40 mL/kg in the evening. Feeding amounts increased by 20 mL/kg/d until reaching 200 mL/kg/d by postnatal day 5 (P5) and thereafter. An intramuscular vitamin K injection of 0.25 mg/kg was given at P1, and a mixture of probiotics and vitamins (Col-O-Cat from SanoBest, Hertogenbosch (Den Bosch), The Netherlands) was given enterally at P2 and P3. Kittens were removed from the incubator for weighing, KYC administration, and tube feeding.

### 4.2. Animal Treatment

Kittens were randomly assigned by sex to the room air (NOX) or hyperoxia (HOX, 95% O_2_) group after the first feed. Half of the kittens received daily intraperitoneal injections of 5 mg/kg KYC or an equal volume of PBS as a control. Health status was checked twice daily after removal from the incubator and under a heating lamp, for weighing, injections, and tube feeding, each lasting less than 15 min. In-house veterinarians checked on the kittens daily.

### 4.3. Histology

The lungs were perfused with normal saline through the right ventricle after the left atrium was perforated. The left lungs were inflated by 10% neutral-buffered formalin at 20 cm-H_2_O (1.9 kPa) for 1 h after the left bronchi were cannulated for histology. The left bronchi were securely tied with surgical silk after pressure inflation, fixed in 10% neutral-buffered formalin for 24 h, and then embedded in paraffin. Lung paraffin sections (5 μm) were mounted on SuperFrost plus-coated slides (Denville Scientific, Metuchen, NJ, USA). After deparaffinization, sections were stained with hematoxylin and eosin (H&E). Images, devoid of major bronchi and large blood vessels, were systematically captured using a programmable Keyence BZ-X710 All-in-One Fluorescence Microscope. Mean linear intercept (MLI) was used to estimate the volume-to-surface ratio of acinar airspaces, and the radial alveolar count (RAC) was used to assess alveolar structure complexity [44].

Quantified data for MLI and RAC were obtained as we previously reported [45]. For MLI, each rabbit lung had six randomly obtained images. We overlaid eight evenly spaced 600 µm (L) lines on each image and counted how many alveolar walls intersected with each line (I). The MLI of each line was calculated as L/I. The average of the eight L/Is was used as MLI for the image. The average of the MLIs obtained from six images was chosen for downstream MLI analysis. For the RAC, we first identified respiratory bronchioles and drew a perpendicular line from the center of this bronchiole to the nearest connective tissue septum or pleura. The number of alveolar sacs intersected by each line was recorded and averaged for each image. The average of the six images was used for downstream RAC analysis. The septal wall thickness (SWT) was obtained using the “Local Thickness” plugin in Fiji. For all histologic and morphometric endpoints, the individual animal was the statistical unit; image-level measurements were averaged within each animal before group-level statistical comparisons.

Inflammatory cells were identified by a positive MPO stain on IHC and counted at 40× magnification. The percent medial wall thickness was obtained by using the equation “100 × 2 × Medial Wall Thickness/External Diameter” [46]. Images of the entire left lung section stained with H&E were scanned using the NDP.view2 system. Pulmonary resistance arteries with a diameter of 40–100 µm were used in the downstream calculations. The average of all qualified measurements (*n* > 5) from the lung section was used for comparison. IHC for MPO (+) myeloid cells was performed using the Abcam anti-MPO antibody. Six randomly obtained images under 40× magnification were used for statistical analysis.

### 4.4. Immunoblots

Lung lysates were prepared from the right lungs in MOPS buffer (20 mM 3-*N*-morpholino-propanesulfonic acid, 2 mM EGTA, 5 mM EDTA, 30 mM sodium fluoride, 10 mM β-glycerophosphate, 10 mM Na pyrophosphate, 2 mM Na orthovanadate, 1 mM PMSF, and 0.5% NP-40 at pH 7.0) by Bullet Blender (Next Advance, Inc., Averill Park, NY, USA). For Western blotting analysis, 30 μg of lysate protein was resolved by SDS-PAGE, transferred to nitrocellulose membranes (0.22 µm), and then probed with appropriate primary antibodies overnight at 4 °C. Signals were generated after incubation with horseradish peroxidase-conjugated goat anti-rabbit (1:10,000) using Pierce™ ECL Western Blotting Substrate (#32209, Thermo Scientific™, Rockford, IL, USA) and recorded by an iBright FL1000 Imaging System (Invitrogen™, Carlsbad, CA, USA). ImageJ 1.54t-processed integrated optical density (IOD) and β-actin signal were used as loading controls.

### 4.5. Statistical Analysis

Data were analyzed using GraphPad Prism version 11.0.0 for Windows (GraphPad Software, San Diego, CA, USA) or R (Version 4.5.1). The distribution of the data was assessed using the Shapiro–Wilk test. Unpaired *t*-tests or the Mann–Whitney U test were used to compare two groups based on the data distribution determined by the Shapiro–Wilk test. Welch’s test was used when a significant difference in variance between two groups was detected. In comparison, one-way ANOVA with Tukey post hoc test after Levene’s test for equality of error variances was used to compare data between more than two groups. Kruskal–Wallis test with Conover–Iman post hoc test was used for data with skewed distributions. All *p*-values were corrected for multiple comparisons. Two-way ANOVA was performed to compare weight differences among the four groups. Values are expressed as the mean ± SD, and scatter plots are shown in the figures. Data on term rabbit kittens were shown as a reference and were not included in statistical analyses. The mortality rates were compared using the log-rank Mantel–Cox and Gehan–Breslow–Wilcoxon tests tests. *p*-values <0.05 were considered statistically significant.

## 5. Conclusions

The present findings extend prior observations from neonatal rat studies into a developmentally relevant premature rabbit model. The ability of this model to recapitulate MPO-associated pathology and KYC-associated therapeutic responses supports its value in evaluating candidate BPD therapies. It underscores the need for future studies that incorporate lung function, gas exchange, and direct measurements of pulmonary hypertension.

## 6. Patents

1. SYSTEMS CHEMICO-PHARMACOLOGY DRUGS AND METHODS OF USE.
  Kirkwood Arthur Pritchard, Jr.; Dustin Paul Martin; Ru-Jeng Teng; Billy W. Day; and Stephen Naylor. Pub. No.: US 2022/0409692 A1 (NON-PROVISIONAL FULL APPLICATION).

2. DUAL ROLE COMPOUNDS WITH PRODRUG AND SYSTEMS CHEMICO-PHARMACOLOGY DRUG PROPERTIES. Billy W. Day; Kirkwood Arthur Pritchard, Jr.; Ru-Jeng Teng; and Stephen Naylor. FILED 04/21/2025 US PROVISIONAL PATENT APPLICATION 63/791,992.
3. SYSTEMS CHEMICO-BIOPROBES DERIVED FROM SYSTEMS CHEMICO-PHARMACOLOGY DRUGS FOR TARGET DISCOVERY AND COMPANION DIAGNOSTIC APPLICATIONS. Kirkwood Arthur Pritchard, Jr.; Billy W. Day; Ru-Jeng Teng; and Stephen Naylor. FILED 04/21/2025 US PROVISIONAL PATENT APPLICATION 63/792,015.

## Data Availability

The original contributions presented in the study are included in the article; further inquiries can be directed to the corresponding author.

## Abbreviations

The following abbreviations are used in this manuscript:

BPD	Bronchopulmonary dysplasia
DAMP	Damage-associated molecular patterns
ECL	Enhanced chemiluminescence
Hmgb-1	High Mobility Group Box 1
HOX	Hyperoxia
HPF	High power field
IHC	Immunohistochemistry
IOD	Integrated optical density
KYC	N-Acetyl-lysyltyrosylcysteine amide
MLI	Mean linear intercept
MPO	Myeloperoxidase
NOX	Normoxia
NZW	New-Zealand White
PBS	Phosphate-buffered saline
RAC	Radial alveolar count
TFA	Trifluoroacetic acid
SWT	Septal wall thickness
UPR	Unfolded protein response
